# Pneumorrhachis Secondary to a Sacral Decubitus Ulcer

**DOI:** 10.5811/westjem.2016.4.30296

**Published:** 2016-06-13

**Authors:** Siamak Moayedi, Lisa Babin

**Affiliations:** * University of Maryland School of Medicine, Department of Emergency Medicine, Baltimore, Maryland; † University of Maryland, School of Medicine, Baltimore, Maryland

## Abstract

An elderly woman with a chronic decubitus sacral ulcer presented to the emergency department with sepsis. A computed tomography of her abdomen showed diffuse gas extending throughout the thoracolumbar spinal canal. Pneumorrhachis is a rare radiographic finding defined as gas within the spinal canal. There are many causes of pneumorrhachis ranging from trauma to infection. In this case the pneumorrhachis was caused by direct spread of gas-forming organisms from vertebral osteomyelitis. Emergency physicians should know about the implication of gas in the spinal canal in the setting of sepsis.

## INTRODUCTION

In this case report, we describe our emergency department (ED) care of an elderly woman with a chronic decubitus sacral ulcer associated with sepsis and meningitis. A computed tomography (CT) of her abdomen showed gas in her thoracolumbar spinal canal. Pneumorrhachis, a rare radiographic finding, is defined as gas within the spinal canal. In this case, the pneumorrhachis was caused by direct spread of gas-forming organisms from vertebral osteomyelitis. Causes and suggested therapeutic approaches are discussed.

## CASE REPORT

A 76-year-old woman with multiple sclerosis and diabetes mellitus was transported to the ED by ambulance from her private residence. Her family had called 9-1-1 because they had perceived a change in her mental status. Over the course of two days, the patient had become non-verbal and the family had noticed intermittent episodes of “arm spasms” followed by prolonged periods of unresponsiveness. At baseline, the patient was fully alert and oriented and capable of coherently communicating. She had been chronically bed-bound because of paralysis of her lower extremities. During the past three months, a large bed sore had developed on her sacral area.

The patient’s vital signs were significant for hypothermia (95.7F rectal) and tachycardia (109 beats/min). Her blood pressure, respiratory rate and pulse oximetry were within normal limits. Her bedside serum glucose concentration was elevated (327mg/dL).

Physical examination revealed an elderly woman mumbling incoherently. She did not follow any commands. Her mucus membranes were dry. Her neck was supple. Her heart and lung exams were unremarkable beyond the tachycardia. She had diffuse abdominal discomfort with palpation, indicated by facial grimacing. Her lower extremities were atrophied and contracted. Examination of her back revealed a large stage 4 decubitus ulcer extending from her sacrum to her lower lumbar spine. There was purulent and malodorous discharge with surrounding cellulitis of the wound edges.

The patient was assessed to be septic and was started on broad-spectrum antibiotics (piperacillin/tazobactam and vancomycin). She was hydrated with two liters of normal saline.

Initial laboratory tests included two sets of blood cultures, a complete blood count and a basic metabolic panel. Results showed a white blood cell count of 34.6K/cm^2^, hemoglobin of 10.7gm/dL, and platelet count of 540K/cm^2^. Her basic metabolic panel showed the following concentrations: sodium 137mEq/L; chloride 88mEq/L; potassium 4.1mEq/L; bicarbonate 26mEq/L; blood urea nitrogen 23mg/dL; creatinine 0.80mg/dL; and glucose 298mg/dL. A chest radiograph was unremarkable.

**Figure 1:**
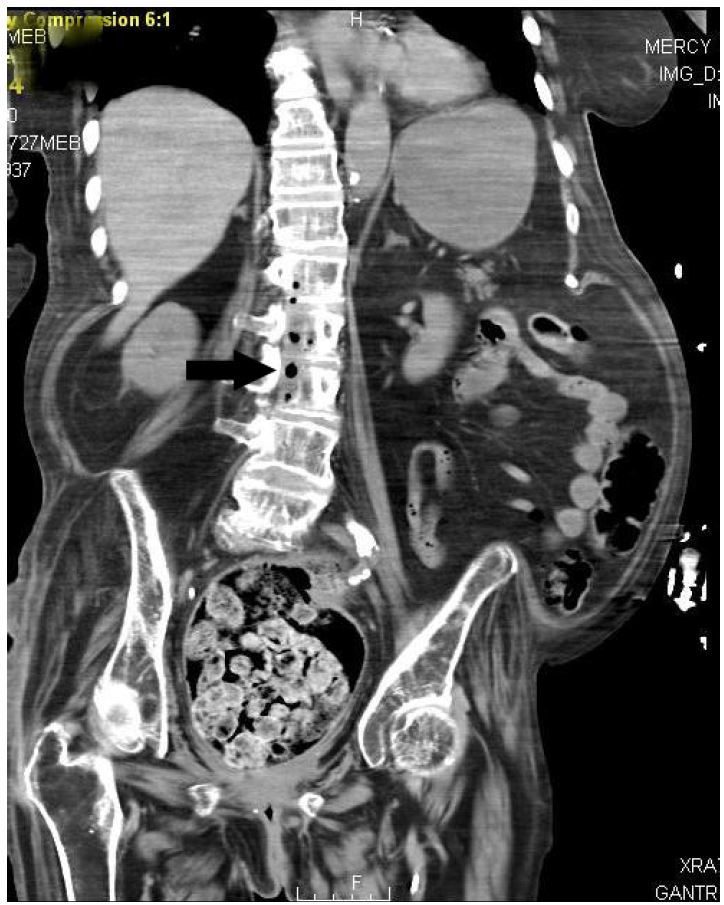
Computed tomography reconstructed coronal image of the abdomen and pelvis demonstrating gas in the thoracolumbar spinal canal (arrow).

**Figure 2:**
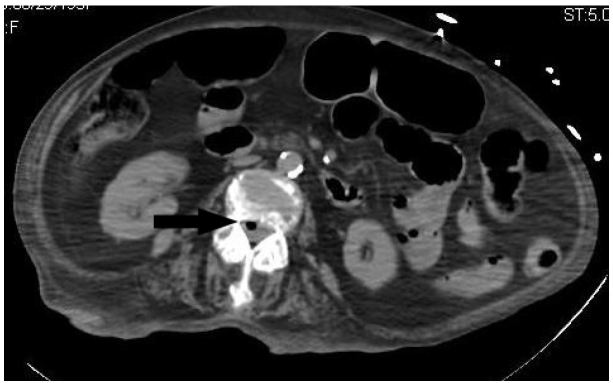
Computed tomography axial image of the abdomen demonstrating gas in the thoracic spinal canal (arrow).

Within one hour after her arrival in the ED, the patient had another episode of “arm spasm” that lasted less than one minute. It was reported by the family members who were with the patient at the time but was not witnessed by medical personnel. This event was presumed to be a seizure, so the patient was intubated to protect her from possible aspiration and prevent hypoxia. Given her apparent abdominal tenderness on physical examination, the presumed seizure, and her altered mental status, CTs of her abdomen and brain were ordered. The abdominal CT (Figures 1 and 2) showed diffuse gas extending throughout the thoracolumbar spinal canal. A small amount of subcutaneous emphysema was noted near the sacrum, and the radiologist was concerned about sacral bone osteomyelitis. Her brain CT showed gas within the right frontal horn and the subarachnoid spaces of the craniocervical junction.

Levetiracetam was administered for seizure prophylaxis, and the initial empiric antibiotic coverage was augmented with cefepime and metronidazole for improved cerebrospinal fluid (CSF) penetration and anaerobic bacterial coverage. Her family refused a lumbar puncture. The patient was admitted to the intensive care unit (ICU) with a diagnosis of sepsis, meningitis, and infected sacral ulcer.

Blood cultures were negative for growth, but a sacral wound culture grew fecal flora. After 36 hours in the ICU, the patient’s condition had not improved, and the family requested comfort measures for her. She died six days after initial presentation.

## DISCUSSION

Pneumorrhachis, a rare radiographic finding, is defined as gas in the spinal canal.^1^,^2^ Gordon and Hardman first reported this phenomenon in 1977.^1^ Since then, it typically has been described as a result of trauma^2^–^4^ or spine surgical procedures^2^ or in connection with other conditions such as pneumomediastinum.^1^–^7^ In our review of the literature, there are limited case reports of pneumorrhachis linked to an infectious disease process including epidural abscess,^6^ hematogenous spread of intraperitoneal sepsis,^8^ and as a complication of decubitus pressure ulcer.^9^

Pneumorrhachis can be iatrogenic (usually a result of spine surgery or lumbar puncture), traumatic (both penetrating and blunt), or non-traumatic (resulting from inhalation drug abuse or invasive tumor progression or from a spontaneous mechanism such as a violent coughing fit).^2^ Half of the 71 reported cases identified in a literature review by Oertel and colleagues were the result of trauma.^2^

Most patients with an incidental finding of pneumorrhachis on CT imaging are asymptomatic. Infection must be on the differential diagnosis for any septic patient with a CT demonstrating pneumorrhachis, especially in the absence of trauma. Infectious pneumorrhachis can be caused by hematogenous spread, as described by Amit et al,^8^ but can also be a direct extension of a local process, such as vertebral osteomyelitis caused by a gas-forming organism. Concomitant pneumocephalus might also be seen in these patients, as in our case.^5^,^8^,^9^

Because of the rare nature of pneumorrhachis as well as its variety of causes, no standard guidelines exist as to its management. Patients with traumatic or surgical pneumorrhachis are typically asymptomatic and are thus managed conservatively with a high concentration of supplemental oxygen, which aids in the redistribution of air back into the bloodstream.^1^–^8^ In some instances, surgical intervention is required to relieve spinal compression or correct a fistula.^2^,^7^ If an infectious process is suspected, lumbar puncture should be performed whenever possible to culture for the responsible organism. Broad-spectrum antibiotics that can cross the blood-brain barrier should be initiated as soon as possible and should include coverage for anaerobic bacteria.

Infectious pneumorrhachis is a rare but important CT finding. Emergency physicians should know about the significance and implication of gas in the spinal canal in the setting of sepsis. Furthermore, in the evaluation of septic and delirious patients with potential osteomyelitis of the spine, meningitis caused by direct extension into the spinal canal should be considered.
